# Cashew apple juice supplementation enhanced fat utilization during high-intensity exercise in trained and untrained men

**DOI:** 10.1186/1550-2783-10-13

**Published:** 2013-03-07

**Authors:** Piyapong Prasertsri, Thapanee Roengrit, Yupaporn Kanpetta, Terdthai Tong-un, Supaporn Muchimapura, Jintanaporn Wattanathorn, Naruemon Leelayuwat

**Affiliations:** 1 Department of physiology, Faculty of Medicine, Khon Kaen University, Khon Kaen, 40002, Thailand; 2 Graduate School, Khon Kaen University, Khon Kaen, 40002, Thailand; 3 Exercise and Sport Sciences Development and Research Group, Khon Kaen University, Khon Kaen, 40002, Thailand

**Keywords:** Vitamin C, Leucine, Endurance, Fat oxidation rate, Athletes

## Abstract

**Background:**

Exercise training is known to increase fat utilization during exercise. Diets containing antioxidants and branch chain amino acids (BCAAs) are also reported to have potential effects on fat utilization. Cashew apple juice (CAJ) comprises many nutritional components including vitamin C and BCAAs. This study aimed to investigate the effect of CAJ supplementation on substrate utilization during high-intensity exercise in trained and untrained subjects.

**Methods:**

Ten trained and ten untrained men were randomly supplemented with either placebo (PLA) or CAJ at 3.5 ml/kg body mass (BM) /day for 4 weeks with a 4-week washout between treatments in a randomized cross-over design. Before and after the 4-week supplementations all subjects performed cycling exercise at 85% of maximal oxygen consumption for 20 minutes. At rest, before, and immediately after the exercise, venous blood samples were taken to determine glucose, insulin and lipid concentrations. Expired air was collected during the 20 minutes of exercise to calculate substrate utilization.

**Results:**

During the exercise in both trained and untrained groups, there were lower carbohydrate (CHO) and higher fat oxidation rates and contributions to total energy expenditure after the CAJ supplementation compared to the PLA supplementation (p<0.05). These values were greater in the trained group than the untrained group except CHO oxidation rates (p<0.05), which were not significantly different. Moreover, in both trained and untrained groups, resting plasma vitamin C concentrations were significantly higher after the CAJ supplementation compared to the PLA supplementation, without any change after the PLA supplementation. These values were greater in the trained group than the untrained group (p<0.05). There were no significant differences in glucose, insulin or lipid concentrations between the groups’ blood samples.

**Conclusion:**

The findings of this study suggest that CAJ supplementation enhanced fat oxidation during exercise may enhance endurance performance, but specific studies are needed to assess this possibility.

## Background

Carbohydrate (CHO) plays a major role as an energy source for active muscle during high-intensity exercise [1]. Moreover, the increased capacity of fat utilization is known to improve exercise capacity [2]. Therefore, an intervention which increases fat utilization may be important for endurance of athletes. Diet and exercise training are known to increase fat utilization during exercise [3]. It is not known whether this can be enhanced further by dietary supplement interventions which increase fat oxidation in untrained individuals.

Endurance training has been shown to improve fat utilization [4]. Possible mechanisms proposed by a recent study involve changes in fatty acid transport protein content in whole muscle (FAT/CD36 and FABPpm), sarcolemmal (FABPpm) and mitochondrial (FAT/CD36) membranes in female human skeletal muscles [5].

Diets containing antioxidants and branch chain amino acids (BCAAs) are reported to have potential effects on fat utilization [6,7]. The antioxidant, vitamin C is perhaps one of the most widely used vitamins in the world today. Johnston *et al*. [6] reported that vitamin C is important for fat oxidation. This may be due to ascorbic acid (vitamin C) being a co-factor for the biosynthesis of carnitine, a molecule required for fatty acid oxidation [8]. This may contribute to increased utilization of fatty acids in triglycerides as a fat source for muscle contraction, resulting in lower serum triglyceride levels [9]. Leucine, the most utilized BCAA, was found to enhance fat oxidation in obese animals and overweight or obese subjects [10,11]. De Araujo *et al*. [12] showed that supplementation with BCAAs (i.e. leucine, isoleucine, or valine) increases hepatic and muscle glycogen concentrations in exercised rats, suggesting greater fat utilization during exercise [7]. A previous study, however, reported an opposite result [13]. This discrepancy may be due to a different nutritional status, as the rats in Cheng’s study were leucine deficient whereas animals or subjects in other studies were supplemented with leucine. Cheng’s study reported that leucine deficiency increased triglyceride lipolysis, leading to increased fat mobilization via cAMP-PKA-HSL in white adipose tissue [13]. This was supported by the results of upregulation of AdrB3 expression, of AdrB3, the main isoform of β-adrenoceptors in the adipose tissue [13]. Together with the effects on energy expenditure (EE) enhancement in brown adipose tissue and lipogenesis suppression, the leucine deficiency contributed to fatty acid mobilization, resulting in increased fat loss.

Cashew apple is a product of cashew nut manufacturing. It is popularly consumed in the form of juice which comprises many nutritional components, including vitamin C and BCAAs [14,15]. For this study it was hypothesized that cashew apple juice (CAJ) would further enhance fat oxidation during high-intensity exercise, adding to the effects of training. Therefore, the effect of CAJ supplementation on substrate utilization during high-intensity exercise in trained and untrained subjects was investigated.

## Materials and methods

### Participants

Ten trained and ten untrained men ages 23 to 33 years old participated in this study. Trained participants performed regular exercise of at least 60 minutes of moderate exercise/day, 5 days/week. They were informed of their role in this study both verbally and in writing before signing a consent form to participate. The consent form was approved by the Human Ethical Committee of Khon Kaen University (HE531365) in accordance with the 1964 Declaration of Helsinki. Subjects partook in a preliminary screening of their blood chemistry and completed health questionnaires and physical examinations before enrolling in the study. None of the subjects was a smoker or had cardiovascular, renal, neuromuscular, orthopedic, or liver disease.

### Power calculation

The sample size of this study was calculated by the WINPEPI program by using the study of Johnston and coworkers from 2006, which reported that marginal vitamin C was associated with fat oxidation rate at rest and during submaximal exercise. It was decided to require 80% power at a significance level of 0.05. Thus, the proposed size was 10 subjects per group and the expected SD was 0.46 kcal/kgBM.

### Study design

The present research was a placebo (PLA)-controlled randomized crossover investigation. Subjects were blinded as to the composition of the CAJ and PLA or which supplement they were on at which times.

### Preparation of CAJ and PLA

The CAJ was provided by the Srisupphaluck Orchid Co., Ltd., Phuket, Thailand. They have been a well-known trader of cashew product for over 50 years. The CAJ consisted of vitamin C (3.36 mg/100 g), leucine (1.64 mg/100 g), isoleucine (3.04 mg/100 g), and valine (0.19 mg/100 g) and had a a total sugar content of 69.8 g/100 mL as measured by the Central Laboratory (Thailand) Co. Ltd., Thailand. The PLA was prepared with a total sugar content equal to that of the CAJ.

### Baseline measurements

Before the experiment, all participants received a routine medical examination from which a medical history was taken and anthropometric measurements of body height, body mass, body mass index, and body composition were made. Body composition was directly measured in the supine position by Dual emission X-ray absorptiometry (DEXA). Fat distribution was indirectly measured by the ratio of waist and hip circumferences. The waist circumference was measured at the end of a normal expiration and at the mid-point between the bottom rib and the superior iliac spine. Hip circumference was measured on a horizontal plane at the site of maximum extension of the buttocks [16].

### Study procedure

Subjects participated in 5 visits, starting with an incremental exercise test to determine maximal oxygen consumption ($VO_{2,\max}$) in trained men and peak oxygen consumption ($VO_{2,\mathrm{peak}}$) in untrained men. One week later, they randomly performed the experiment, consisting of two 4-week phases with a 4-week washout between the treatments. In the experimental phases they were supplemented with either PLA or CAJ (3.5 ml/kg BM/day) continuously for 4 weeks. Before and after each phase, they performed high-intensity exercise by cycling at 85% $VO_{2,\max}$ for 20 min in trained subjects and 85% $VO_{2,\mathrm{peak}}$ in untrained subjects. They fasted overnight before each exercise session. The final dose of CAJ / PLA was taken the day before the exercise session after each phase. Venous blood samples were taken before and after the exercise to determine glucose, insulin and vitamin C concentrations and lipid profile, including total cholesterol (TC), high-density lipoprotein (HDL), low-density lipoprotein (LDL), and triglycerides (TG). During the exercise sessions, expired-air samples were collected to determine substrate utilization (CHO and fat oxidation rates and CHO and fat contribution to total EE) and EE. Throughout the experimental period the subjects were instructed not to change their diets or exercise routines.

### Incremental

$VO_{2,\max}$

 or

$VO_{2,\mathrm{peak}}$ exercise test

Subjects began the test by warming up with free workload (0 watt) cycling for 2 minutes. They then started with a workload at 30–50 watts depending on their fitness status. Workloads were increased by 20–30 watts every 3 minutes until they reached the criteria establishing $VO_{2,\max}$ or $VO_{2,\mathrm{peak}}$; included possession of maximum symptoms of dyspnea (9-10) and fatigue (18-20), determined by rating of perceived dyspnea (RPD) and rating of perceived exertion (RPE) scales; inability to maintain a cycling speed of at least 60 rpm; an increase of heart rate (HR) to predicted HR_max_ (220 - age); and steady or falling ***V***O_2_. Expired-air samples, oxygen saturation, and HR were recorded throughout the test, and the dyspnea and fatigue symptoms were inquired of the subjects at the end of each workload. Electrocardiography was monitored throughout the exercise experiments.

### Measurement of substrate utilization

Expired air was obtained throughout the exercise and was analyzed by a gas analyzer (PowerLab 8/30 ADInstruments, Australia) to determine EE, oxygen consumption ($VO_{2}$), carbon dioxide production ($VCO_{2}$) and respiratory exchange ratio (RER). $VO_{2}\;\left(L/\min\right)\;\mathrm{and}\;VCO_{2}\;\left(L/\min\right)$ (L/min) were used to calculate substrate oxidation rate (g/min) by using the Peronnet and Massicotte equation [17]:

$$\mathrm{CHO}\;\mathrm{oxidation}\;\mathrm{rate}=4.585\;VCO_{2}-3.226\;VO_{2}$$

$$\mathrm{Fat}\;\mathrm{oxidation}\;\mathrm{rate}=1.695\;VO_{2}-1.701\;VCO_{2}$$

### Biochemical assay

Blood samples were collected from the antecubital vein and immediately transferred into EDTA-treated tubes. The tubes were then centrifuged at 3,000 *g* for 10 minutes to remove red blood cells and recover serum. The serum obtained was used to analyze TC, HDL, LDL, TG, and glucose levels using standard automated laboratory methods (Roche Integra 800, Basel, Switzerland) and to analyze insulin by using the radioimmunoassay technique. These methods are routinely used in Srinagarind Hospital, Faculty of Medicine, Khon Kaen University. The plasma was used to analyze vitamin C levels with using Zhang’s method [18].

### Statistical analyses

Data were analyzed using the SPSS statistics software package, version 13. Differences between supplements and groups were tested by two-way analysis of variance (repeated measurement). All data are expressed as means ± SD except when stated elsewhere. All differences are considered significant at *P*< 0.05.

## Results

Baseline anthropometric and physiological parameters of all subjects are shown in Table 1. The trained group had significantly higher values of $VO_{2,\max}$ and work rate_max_ and lower values of fat percentage and fat mass than the untrained group.

**Table 1:** Anthropometric and physiological characteristics of subjects

	**Untrained group (n = 10)**	**Trained group (n = 10)**	***P *value**
Age (yr)	20 ± 2.7	21 ± 1	*NS*
Body mass (kg)	67.7 ± 14.2	67.2 ± 10.2	*NS*
Height (m)	1.69 ± 0.1	1.72 ± 0.1	*NS*
BMI (kg/m^2^)	23 ± 3.0	22.7 ± 2.4	*NS*
Waist circumference (cm)	75.3 ± 10.5	75.5 ± 4.7	*NS*
Hip circumference (cm)	94.9 ± 8.1	93.3 ± 4.9	*NS*
W/H ratio	0.79 ± 0.1	0.81 ± 0.3	*NS*
Body fat (%)	21.9 ± 8.1	16.2 ± 6.6	*NS*
Fat mass (kg)	14.3 ± 5.6	13.8 ± 8.3	*NS*
Fat free mass (kg)	51.4 ± 5.8	53.4 ± 5	*NS*
$VO_{2,\mathrm{peak}}$ (ml/kgBM/min)	31.2 ± 8.5	45.6 ± 4.1	*0.000*
$VO_{2,\max}$ (ml/kgBM/min)			
$VO_{2,\mathrm{peak}}$ (ml/kgFFM/min)	41.2 ± 9.3	58.5 ± 4.9	*0.000*
$VO_{2,\max}$ (ml/kgFFM/min)			
Work rate_max_ (watts)	136 ± 14.3	178 ± 13.9	*0.000*

Values are mean ± SD, n = 10 in each group. BM, body mass; BMI, body mass index; W/H, waist to hip circumference ratio; $VO_{2,\mathrm{peak}}$, peak oxygen consumption; $VO_{2,\max}$, maximal oxygen consumption; FFM, fat free mass; Work rate_max_, maximal work rate.

*P* value, significantly different from the untrained group; *NS*, not significant.

**Figure 1:**
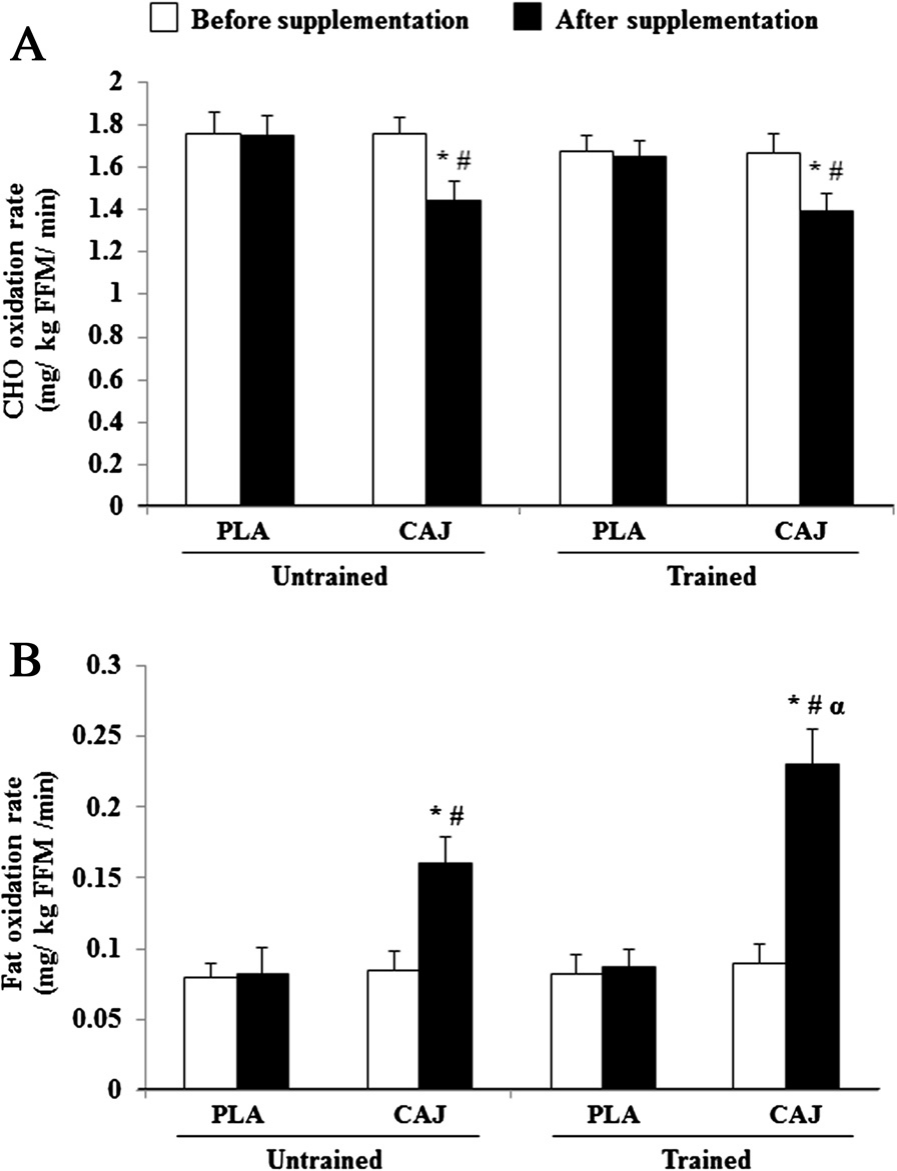
**CHO (A) and fat (B) oxidation rates during exercise at 85% **$VO_{2,\max}$**or**$VO_{2,\mathrm{peak}}$**after 4-week placebo (PLA) and cashew apple juice (CAJ) supplementation.** Values are mean ± SE, n = 10 in each group. CHO, carbohydrate. * Significantly different from before supplementation, p<0.05, # significantly different from the PLA group, p<0.05, α significantly different from the untrained group, p<0.05.

**Figure 2:**
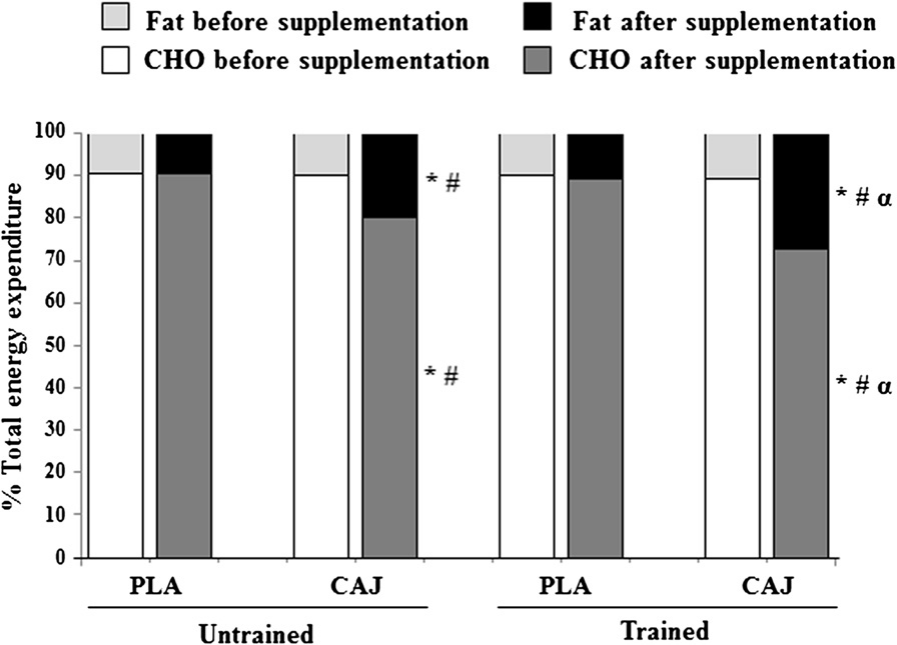
**Relative contribution of substrate to total energy expenditure during exercise at 85%**$VO_{2,\max}$**or**$VO_{2,\mathrm{peak}}$**after 4-week placebo (PLA) and cashew apple juice (CAJ) supplementation.** Values are mean, n = 10 in each group. * Significantly different from before supplementation, p<0.05, # significantly different from the PLA group, p<0.05, α significantly different from the untrained group, p<0.05.

Before and after both supplementation periods, the trained group had significantly higher $VO_{2,\max}$, total EE, work rate_max_ and work rate 85% $VO_{2,\max}$ and lower fat mass than the untrained group, without any differences in percentage of $VO_{2,\max}$, HR_max_, RER, RPD, RPE, or HR during exercise. Interestingly, the trained group showed a greater fat oxidation rate than the untrained group only after the 4-week ingestion of the CAJ (0.23 vs 0.16 g/min; p<0.05) (Figure 1). There were no significant differences in CHO oxidation rates between the trained and untrained groups after the 4-week ingestion of the CAJ. In the untrained group, the contributions of CHO and fat to total EE during exercise were lower and higher, respectively, after the CAJ supplementation than after taking the PLA supplementation (80 vs 90%; p< 0.05 and 20 vs 10%; p< 0.05) (Figure 2). In the trained group, the contributions of CHO and fat to total EE during exercise were also lower and higher, respectively, after CAJ supplementation than after taking the PLA (73 vs 89%; p<0.05 and 27 vs 11%; p<0.05) (Figure 2).

**Figure 3:**
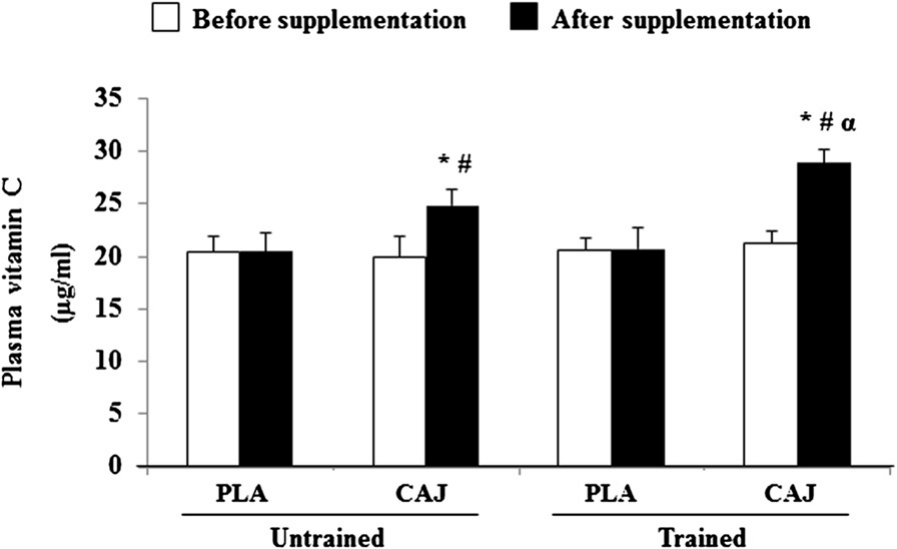
**Plasma vitamin C concentration immediately after exercise at 85%**$VO_{2,\max}$**or**$VO_{2,\mathrm{peak}}$**after 4-week placebo (PLA) and cashew apple juice (CAJ) supplementation.** Values are mean ± SE, n = 10 in each group. * Significantly different from before supplementation, p<0.05, # significantly different from the PLA group, p<0.05, α significantly different from the untrained group, p<0.05.

In both the trained and untrained groups, resting plasma vitamin C concentrations were significantly increased after the CAJ supplementation (p<0.05) without any change after receiving the PLA (Figure 3). There were significantly higher vitamin C concentrations after the CAJ supplementation than the PLA administration (p<0.05). CAJ supplementation, however, had no effect on the metabolic profiles taken at rest and after exercise sessions, including serum glucose, insulin, TC, TG, HDL, or LDL, in either the trained or untrained subjects. With the PLA administration, there were also no significant changes in any parameters over the 4-week treatment period in either the trained or untrained subjects.

## Discussion

This study showed that the 4-week CAJ supplementation increased fat contribution and decreased CHO contribution to total energy expenditure during high-intensity exercise in both the trained and untrained subjects, with a greater change in the trained subjects.

It should be noted that this study assessed whole-body substrate utilization. Therefore, the changes in specific sources of energy used cannot be defined. Normally, at high-intensity exercise, oxidation rates of muscle glycogen and plasma glucose increase and those of both plasma free fatty acids and triacylglycerol fat sources (sum of intramuscular plus lipoprotein-derived triacylglycerol) decrease [1]. The reduction in fat oxidation is most likely due to a downregulation of carnitine palmitoyltransferase I, which may be due to a decline in intracellular free carnitine availability or pH. The supplementation with CAJ may enhance fat oxidation via the effect of one of its constituents, vitamin C [6,7], on carnitine synthesis [19]. Vitamin C acts as a co-factor for two necessary enzymes, ε-N-trimethyl-L-lysine hydroxylase and γ-butyrobetaine hydroxylase, which are required for the biosynthesis of carnitine [20,21], an important co-factor in fat oxidation in skeletal muscle [8].

In addition, leucine, another constituent of CAJ, appears to have considerable effects on energy metabolism [10,11,22]. It induced a significant increase in fat oxidation in C2C12 muscle cells [22] and rats [10] via an improvement in mitochondrial oxidative function. Leucine also affects adipose tissue, reducing fatty acid synthase expression in human adipocytes [11]. A previous study showed that supplementation with leucine increases hepatic and muscle glycogen concentrations immediately after exercise [12] suggesting greater fat use during exercise [7].

The current study did not find any changes in blood glucose and lipids, which are also energy sources for active muscle during exercise. The unaltered concentrations of blood glucose after the supplementation of CAJ in this study may be because subjects were healthy. During exercise, blood glucose concentration must be maintained by hepatic glycogenolysis and gluconeogenesis, as they are energy sources for the brain [23]. Increases in glucagon and catecholamine are apparently responsible for such maintenance [24].

Another component of CAJ, the anacardic acids [25], are worth considering but were not analyzed in this study. Dietary anacardic acids at 0.1% w/w have been shown to decrease body fat deposition in rat liver, possibly due to an uncoupling action of the anacardic acids on mitochondrial oxidative phosphorylation [26]. If such a mechanism functions in human subjects, it may contribute to the increased fat utilization after the ingestion in CAJ of this study.

The enhanced fat oxidation rate in this study could be beneficial for endurance performance by providing energy for the muscle and sparing intramuscular glycogen for possible use in the later stages of competitive sports, e.g., long distance running and swimming.

The enhanced effect on fat utilization during exercise seems to be important for some populations, particularly Thai people. Janyacharoen *et al*. [27] demonstrated that during exercise at all intensities CHO played a more important role as an energy source than fat. This may be a significant reason for the lower endurance capacity of Thais compared to Caucasian athletes, affecting Thai championship status. Therefore, CAJ ingestion has a potential advantage of bringing Thai sport players to success on the scale of world competition. In addition, quantitative measurements of anacardic acids or other antioxidants in the CAJ, e.g., phenolic compounds, will provide more information of other ingredients in the CAJ that may have an effect on lipid metabolism.

## Conclusions

The findings of this study suggest that CAJ enhanced fat oxidation during exercise and may enhance endurance performance, but specific studies are needed to assess this possibility.

## Abbreviations

BCAAs: Branch chain amino acids; CAJ: Cashew apple juice; CHO: Carbohydrate; DEXA: Dual emission x-ray absorptiometry; EDTA: Ethylene diamine tetra acetic acid; EE: Energy expenditure; HDL: High-density lipoprotein; HR: Heart rate; HRmax: Maximum heart rate; LDL: Low-density lipoprotein; PLA: Placebo; RER: Respiratory exchange ratio; RIA: Radioimmunoassay; RPD: Rating of perceived dyspnea; RPE: Rating of perceived exertion; SD: Standard deviation; TC: Total cholesterol; TG: Triglycerides; VCO2: Carbon dioxide production; VO2: Oxygen consumption; VCO2,max: Maximal oxygen consumption; VCO2,peak: Peak oxygen consumption

## Competing interest

No conflict of interest was reported by the authors of this paper.

## Authors’ contributions

NL conceived and designed the study and prepared the manuscript. TT provided medical coverage throughout the experiment. TR and YK carried out all the experimental work and statistical analysis and helped to draft the manuscript. All authors read and approved the final manuscript.
